# Understanding for Which Students and Classes a Socio-Ecological Aggression Prevention Program Works Best: Testing Individual Student and Class Level Moderators

**DOI:** 10.1007/s10964-021-01553-6

**Published:** 2021-12-18

**Authors:** Lisa Bardach, Takuya Yanagida, Petra Gradinger, Dagmar Strohmeier

**Affiliations:** 1 University of Tübingen, Hector Research Institute of Education Sciences and Psychology, Walter-Simon-Straße 12, 72072 Tübingen, Germany; 2 University of Vienna, Faculty of Psychology, Universitätsstraße 7, 1010 Vienna, Austria; 3 University of Applied Sciences Upper Austria, School of Applied Health and Social Sciences, Garnisonstraße 21, 4020 Linz, Austria

**Keywords:** Aggressive behavior, Victimization, Randomized controlled, Moderator, Effectiveness, Climate, Ethnic diversity

## Abstract

School-based aggression prevention programs may not be equally effective for all students and classes, depending on student and class characteristics. This study investigated moderators of a cluster randomized controlled socio-ecological aggression prevention program’s effectiveness (change from pretest to posttest, sample: 2,042 preadolescents, mean age = 11.7 years, *SD* = 0.09, 47.6% girls) and sustainability (change from posttest to follow-up test, sample: 659 preadolescents, mean age = 12.7 years, *SD* = 0.08, 47.9% girls). The program worked better in multicultural classes, as greater ethnic diversity strengthened the program’s effectiveness and sustainability. Moderating effects of a positive social class climate and higher baseline levels of aggressive behavior and victimization were also found. These results advance socio-ecological theorizing and can help develop more contextualized interventions.

## Introduction

Aggressive behavior represents a pervasive problem in childhood and adolescence, with a mean prevalence rate of around 35% for both aggressive behavior as well as victimization across studies and contexts (see Modecki et al., 2014 for a meta-analysis). In addition, aggressive behavior peaks in preadolescence (Inchley et al., 2016). Critically, being the recipient of peer aggression as well as engaging in aggressive behavior oneself have long-term negative consequences, such as mental health problems, chronic diseases, and school maladjustment (e.g., Holt et al., 2015). Reducing aggressive behavior and victimization in schools has therefore become an international priority. From a socio-ecological perspective, aggressive behavior and victimization are understood as complex systemic problems with mechanisms operating on several interacting levels (Bronfenbrenner, 1979; Espelage, 2014). Consequently, a socio-ecological aggression prevention program not only targets individual students, but also assumes that aggressive behavior unfolds in response to contextual characteristics (Gradinger & Strohmeier, 2018). Gaining insight into whether a socio-ecological aggression prevention program leads to differential changes conditional on the characteristics of the participating individual students or entire classes is critical (moderator effects, or aptitude-by-treatment interactions, Preacher & Sterba, 2019). However, research on moderators of the effects of aggression prevention programs is still scarce. To fill this gap, the current study investigated individual student and class level moderators of the effectiveness (i.e., changes in aggressive behavior and victimization from pretest to posttest) and the sustainability (i.e., changes in aggressive behavior and victimization from posttest to follow-up test) of the ViSC Social Competence program. The ViSC program is a cluster-randomized, whole-school socio-ecological aggression prevention program designed for preadolescents that has been implemented at scale and evaluated in several countries during the last decade (e.g., Strohmeier & Spiel, 2019; Strohmeier et al., 2021). In this study, multi-level modeling was employed to test a set of conceptually and empirically relevant potential moderators of the ViSC program’s effectiveness and sustainability, including individual student characteristics (gender, initial levels of aggressive behavior and victimization) and classroom characteristics (positive social class climate, ethnic diversity).

### Aggressive Behavior and Victimization: Types and Interventions

Interpersonal aggressive behavior covers any behavior that is intended to harm or injure another person, who is motivated to avoid that harm (e.g., DeWall et al., 2011). Different types of aggressive behavior can be distinguished. *Physical aggressive behavior* describes any act of physical violence or threats of physical harm towards others, for example by hitting, kicking, or biting them (e.g., Kaye & Erdley, 2011). On the other hand, *relational aggressive behavior* refers to manipulating or harming another’s social standing, for example by means of verbal or nonverbal exclusion, threatening friendship withdrawal, or spreading rumors (e.g., Leff et al., 2010). *Bullying perpetration*, a further subcategory of aggression, captures a harmful relationship between a perpetrator and a target that is characterized by an imbalance of power, repetition, and the perpetrator’s intention to hurt the target (Olweus, 1993). Victimization refers to exactly the same types of behavior, but from the perspective of the targets (i.e., physical victimization, relational victimization, bullying victimization).^1^ The present study followed a broad and comprehensive conceptualization of aggressive and victimization (see also e.g., Ladd et al., 2017). Hence, (a) physical aggressive behavior, relational aggressive behavior, and bullying perpetration were considered as indicators of the overarching aggressive behavior construct, and, (b) physical victimization, relational victimization, and bullying victimization were considered as indicators of the overarching construct victimization.

Several research syntheses have shown that programs designed to counteract or prevent aggression and victimization in schools are effective on average (e.g., Gaffney et al., 2021; Hendriks et al., 2018). In addition to programs’ effectiveness in terms of changing the intended outcomes, their sustained impact (sustainability) beyond the actual intervention period is of interest. However, whereas changes between pretest and posttest assessments (i.e., program effectiveness) have been widely studied, sustained effects of such programs (i.e., program sustainability) requiring additional follow-up assessments have been explored less systematically (but see e.g., Andreou et al., 2008; Palladino et al., 2016). One previous study with follow-up assessment relied on a sample of fourth and sixth grade students (Andreou et al., 2008). Pre- and posttest data was collected at the middle and end of the same school year. The follow-up test took place six months later, during the next school year. The findings indicated positive short-term program outcomes concerning students’ attitudes towards bullies and victims, perceived efficacy of intervening in bully–victim incidents and actual rates of intervening behavior. However, the positive effects were not sustained over the long-term (Andreou et al., 2008). A two-study paper with adolescents in their first year of high school revealed that the intervention significantly predicted changes over time in all target variables, including victimization and bullying. Specifically, the target variables were stable for the control group, but decreased significantly over time for the intervention group. Long-term effects on the follow-up test 6 months later were also found (Palladino et al., 2016). Overall, more research identifying aggression prevention programs’ long(er)-term effects are needed in order to determine whether intervention effects are maintained (i.e., sustained effects), fall back (i.e., fade-out effects), or increase further (i.e., sleeper effects) (van Aar et al., 2017).

### Program Moderators: Differentiated Effects for Different Students and Classes?

Program effectiveness and sustainability are driven by a number of factors, such as program elements and quality of implementation (e.g., Gaffney et al. 2021; Low & van Ryzin, 2014). In addition, a range of further aspects beyond the interventionists’ control can come into play. According to the socio-ecological model of development (Bronfenbrenner, 1979; Espelage, 2014), factors influencing and interacting with aggressive behavior and victimization can be located on several systemic levels. Students may react differentially to programs due to specific individual characteristics they bring to the situation (e.g., Stoltz et al., 2013). At the same time, students find themselves in complex social contexts that can become risk or protective factors for aggression and victimization and can thus also affect an intervention’s functioning (e.g., Espelage, 2014). Accordingly, in order to more thoroughly understand how school-based socio-ecological aggression prevention programs work or fail to work, a focus on both individual student features and contextual features (e.g., schools, classes) as potential program moderators is important (e.g., Hendriks et al., 2018).

#### Individual Student Characteristics

According to socio-ecological perspectives, a person’s development unfolds as a result of ontogenetic characteristics and complex, interrelated interactions at the individual, micro-, meso-, exo-, macro-, and chronosystem levels (Bronfenbrenner, 1979). On the individual level, the socio-ecological model seeks to identify the biological and person-related factors influencing behavior and development. Research on aggression has identified a large number of relevant individual level variables (Gaffney et al., 2021). However, individual level program moderators have only rarely been studied, which limits the current understanding of whether and how program effects depend on specific individual features. To start filling this void in the literature, the present study focused on three individual student characteristics as potential moderators.

First, aggression programs may be most effective in changing students’ engagement in aggressive behavior when the base rates of aggressive behavior are high enough for meaningful changes to be possible (e.g., Wilson et al., 2003). Aligned with this, larger effect sizes for aggression-related outcomes have been obtained for students with relatively higher levels of *baseline aggressive behavior* (i.e., aggressive behavior assessed at pretest, see Hendriks et al., 2018). Similarly, a meta-analysis of aggression interventions revealed that higher-risk youth exhibited greater reductions in aggressive behavior following the intervention (Wilson et al., 2003). Based on prior findings on such so-called risk x intervention interactions for aggressive behavior (see also Juvonen et al., 2016), it seems plausible to assume that higher *baseline levels of victimization* should also facilitate stronger program effects for victimization. In addition, prior intervention research has indicated that students in the intervention group with higher baseline aggressive behavior not only exhibited less aggressive behavior toward peers after the intervention, but also experienced less victimization (DeRosier et al., 2005). As aggressive behavior and victimization have often been found to co-occur within individuals, it is likely that higher baseline levels of aggressive behavior and victimization, respectively, should also enhance program effects on the other type of outcome. That means that stronger changes in victimization may occur in the presence of higher baseline levels of aggressive behavior, and stronger changes in aggressive behavior in the presence of higher baseline levels of victimization.

Second, boys typically report higher levels of both bullying perpetration and victimization and score higher on physical aggression (e.g., hitting, kicking) than girls. Although it has been argued that girls are more likely to be involved in social/relational forms of aggression (e.g., spreading rumors, excluding or ignoring others), only small-to-trivial gender differences in social aggression have been documented (Card et al., 2008). Many aggression prevention programs, including ViSC, aim to foster empathy or defending others using group-based and cooperative methods that are suspected to be more suitable for girls (see e.g., Zych et al., 2019). Nonetheless, research on the moderating effects of gender for aggression intervention programs has produced mixed findings. For instance, the effects of an intervention on child-reported proactive aggression were shown to differ as a function of participants’ gender, with stronger effects for boys (Stoltz et al., 2013; see also Kennedy, 2020). Other studies evaluating the effectiveness of aggression interventions reported that girls profited more (e.g., Ossa et al., 2020), while still others did not find gender to be a statistically significant moderator (see e.g., Hendriks et al., 2018). Testing for potential moderating effects of gender, as a key socio-demographic characteristic, is always relevant, as it clarifies whether the same program is equally adaptive for all participants irrespective of gender; however, the mixed current body of research makes it difficult to derive clear predictions about the role of gender for the current aggression prevention program’s functioning.

#### Classroom Characteristics

Characteristics of the school and class context represent important microsystems that need to be taken into account, as they could conceivably influence an intervention’s effectiveness and sustainability. Interventions do not occur in a vacuum, but are instead embedded in complex social contexts, and contextual features are particularly critical for programs that are delivered to entire classes or schools (e.g., Low & van Ryzin, 2014). A promising moderator candidate that has received much attention in prior research on aggression is the prevailing *school or class climate* (e.g., Elsaesser et al., 2013). School climate has often been conceptualized as a broad, multi-dimensional construct capturing school experiences in different domains, including the academic, community, safety, and institutional environment (Wang & Degol, 2016). In Austria, the context of the current study, students usually stay together in the same class with the same classmates throughout the day and from grade to grade within a given school. Moreover, cross-class peer relations and bullying are scarce in Austria (Gradinger et al., 2012). For these reasons, it is more appropriate to assess climate with reference to the class rather than the school in the Austrian context. This study focused on positive social class climate in terms of a positive cooperative atmosphere in class as a specific aspect of class climate. This aspect most closely resembles features subsumed under the “community” dimension of broader school climate conceptualizations, and was considered particularly relevant for research on moderators of socio-ecological aggression prevention programs like the VisC program, which includes group-based and cooperative methods.

Theoretically, a more positive social climate could facilitate program effects by enhancing adoption, commitment to, and implementation of programs as well as the deployment of skills taught in such programs (e.g., Low & van Ryzin, 2014). Nonetheless, research investigating positive social class or school climate as a moderator of the effects of aggression intervention programs is extremely rare and has been somewhat mixed, as some studies have obtained positive moderating effects (e.g., Dymnicki & Multisite Violence Prevention Project, 2014), whereas others have not (e.g., Low & Van Ryzin, 2014). In light of the convincing theoretical rationale that a positive social climate should matter for the functioning of aggression intervention efforts, more research is clearly needed to test this assumption.

Finally, many societies are becoming increasingly multi-cultural, as are their schools and classrooms (e.g., OECD, 2016). As such, the *ethnic diversity* of classrooms and school settings represents an important contextual characteristic (e.g., Graham, 2018). Ethnic diversity captures the availability of same- vs. cross-ethnic peers in a classroom, which in turn creates varying opportunities for same- vs. cross-ethnic peer relations and interactions, including both friendships and aggression. A class in which the level of ethnic diversity is low comprises only a few students with ethnic backgrounds other than the majority, thus offering low potential for intergroup exchange and peer relations. In contrast, the intergroup peer relation potential is high in an ethnically diverse class, i.e., when students with many different ethnic backgrounds are present. Moreover, in an ethnically diverse class, no cultural group holds a numerical majority position, because there are several smaller ethnic groups present. Thus, ethnic diversity takes the number of ethnic groups as well as their relative proportion in the class into account (Stefanek et al., 2015), whereas simple percentages only capture the relation between a maximum of two groups (e.g., minority vs. majority, non-immigrant vs. immigrant, etc.).

There are strong theoretical reasons to believe that ethnic diversity could act as a protective factor for aggression, as in ethnically diverse classes and schools, the balance of power is unlikely to be tipped in favor of one group over another, and this lack of power differentials may reduce incidents of peer harassment. Accordingly, empirical evidence from the US has shown that students in more ethnically diverse classes felt safer in school and were less harassed by peers (e.g., Juvonen et al., 2018). On the other hand, it has also been argued that ethnic diversity within schools may be a risk factor for aggression. In more diverse settings, cultural differences are likely to exist and may cause frictions or misunderstanding, eventually resulting in aggressive behavior. Some studies conducted in European countries (the Netherlands) have supported the premise of ethnic diversity as a risk factor (e.g., Jansen et al., 2016). In other European countries, no associations were found (e.g., Stefanek et al., 2015). As all of these studies were based on observational designs, whether and how ethnic diversity interacts with the effectiveness and sustainability of programs seeking to prevent face-to-face aggression and bullying is an open yet highly relevant question. This question has never been addressed for even the best researched anti-bullying program (KiVa from Finland) (Yun & Salmivalli, 2021). Some aggression prevention programs, including the ViSC program, involve components known to foster positive intergroup contact between students with diverse backgrounds (e.g., ensuring equal contributions of all students). It may be that such programs work better in more diverse classrooms. Nonetheless, as no prior study has explored moderator effects of ethnic diversity, it is difficult to derive concrete hypotheses.

## Current Study

Given the effectiveness and sustainability of school-based aggression prevention programs may depend on a variety of student and class characteristics, this study sought to address this research gap by analyzing findings from a cluster-randomized, whole-school socio-ecological aggression prevention program. *First*, does the aggression prevention program work as intended, such that aggressive behavior and victimization exhibit a more advantageous trajectory of change over time in the intervention group than in the control group, both from pretest to posttest (Hypothesis 1a, program effectiveness) and from posttest to follow-up (Hypothesis 1b, program sustainability)? *Second*, to ensure that intervention effects are not caused by co-varying individual and class-level characteristics, do findings for program effectiveness and sustainability remain robust when a set of covariates are additionally considered? It was assumed that the effects would not be substantially affected (i.e., not rendered statistically non-significant) by the inclusion of covariates at the class-level (ethnic diversity, positive social class climate) and the individual-level (gender, age, baseline levels of aggressive behavior and victimization). *Third*, do classroom characteristics (positive social class climate, ethnic diversity) interact with the program’s effectiveness and sustainability? It was hypothesized that a more positive social class climate should strengthen program effectiveness (Hypothesis 3a) and sustainability effects (Hypothesis 3b), whereas no a priori hypotheses regarding moderating effects of ethnic diversity were specified and exploratory analyses were conducted. *Fourth*, do individual student characteristics (baseline levels of aggressive behavior and victimization, gender) interact with the program’s effectiveness and sustainability? Stronger intervention effects on changes in aggressive behavior should occur in the presence of higher baseline aggressive behavior and stronger intervention effects on changes in victimization should occur in the presence of higher baseline victimization (Hypothesis 4a for program effectiveness; Hypothesis 4b for program sustainability). Higher baseline levels of aggressive behavior should furthermore moderate program effectiveness and sustainability for victimization and higher baseline levels of victimization should moderate program effectiveness and sustainability for aggressive behavior (Hypothesis 4c for program effectiveness; Hypothesis 4d for program sustainability). No hypotheses for gender as potential moderator were stated and exploratory analyses were carried out.

## Methods

### Design and Procedure

All secondary schools located in the Austrian federal state of Vienna, which is also the capital of Austria, were invited to participate in the ViSC Social Competence program (see next section for a detailed description of the program). Vienna was the federal state for which funding for program implementation and evaluation was available. Of the 155 secondary schools located in Vienna, 34 schools applied to participate. Of these 34 schools, 26 fulfilled the requirements, such as willingness to participate in the accompanying evaluation study. Following a cluster randomization, 13 schools were assigned to the intervention group and five of the 13 remaining schools agreed to serve as control schools.

Table 1 shows that data was collected at three time points (pretest, posttest, follow-up test). At all three time points, students attended lower secondary school and remained with the same classmates. Hence, no school transitions or changes in the classroom composition occurred. The school year in Austria starts in September and finishes at the end of June. The school year is split into two terms, the winter term (September to February), and summer term (February to June), with a longer summer break (July and August). The pre- and posttest were roughly one year apart and took place during the summer semesters of two consecutive school years, while the follow-up test took place six months after the posttest during the winter semester of the third school year. To keep the data collection moths constant, the pretest and posttest were conducted at the end of two consecutive school years (in May and June) during which the ViSC program was implemented. Students were attending either Grade 5 or 6 at pretest and then either Grade 6 or 7 at posttest. Follow-up data collection was scheduled before the exam period in November and December of the third school year, when students were attending either Grade 7 or 8. Due to limited funding, data were collected in November and December and not May and June of the third school year. For the same reason, it was only possible to collect follow-up data from a sub-sample of three intervention and three control schools (see Table 1).

**Table 1:** Study design

	Pretest	Posttest	Follow-up test
Intervention group	10 schools	10 schools	No follow-up data collected
	3 schools	3 schools	3 schools
Control group	2 schools	2 schools	No follow-up data collected
	3 schools	3 schools	3 schools
Total number of schools participating at each measurement point	18 schools	18 schools	6 schools

After permission to conduct the study was obtained from the local school council and school principals, active parental consent was obtained. 71% percent of students were present on the day of data collection for the pretest and had parental consent to participate in the study. Data were collected through internet-based questionnaires. Students completed the questionnaires during one regular school lesson in the school’s computer lab and were supervised by one or two trained research assistants. To avoid any systematic order effects, the order of the items within scales was counterbalanced. Students were assured that their answers would be kept confidential prior to data collection. Although prior studies with the same or overlapping data have already been published, the current study addresses unique research questions by testing moderators of program effectiveness and sustainability for face-to-face aggressive behavior and victimization.

### The ViSC program

The ViSC program is a school-based socioecological social competence program that aims to reduce aggressive behavior and bullying and foster social and intercultural competence among youth aged 11 to 15 (Strohmeier et al., 2012). The program consists of a variety of measures on the school, class, and individual level, and is implemented over one school year (see Strohmeier et al., 2012). It is important to understand that whole-school programs are heterogeneous, as they comprise a variety of different concrete measures and are always implemented in a particular national school system (Gaffney et al., 2021). Thus, the overall effectiveness of whole-school approaches to tackling aggressive behavior also depends on contextual factors. On the school level, the current program aimed to promote teachers’ shared responsibility, meaning that as many teachers as possible have worked out a common understanding of aggressive behavior and bullying, agreed on procedures for how to tackle acute bullying cases, and implemented preventive measures in their classes. On the class level, a 13-unit class project is implemented by teachers in their classes based on a comprehensive manual. The class project’s structure applies principles such as equal status of all students, common goals, cooperation among students, and authority support (e.g., support by teachers), factors that are highly important for fostering positive intercultural relations (Strohmeier et al., 2020). Thus, the program’s intercultural aspect stems from the program structure and instructional methods, while concrete skills and knowledge to reduce aggressive behavior (including bullying) are operationalized via the program content. The units are theoretically based on intergroup contact theory (Allport, 1954), social information processing theory (Crick & Dodge, 1996), and bullying as a group phenomenon (Salmivalli et al., 1996). Students are trained to recognize when something negative is going on in their class, feel responsible for taking action, and react in a way that is likely to improve the situation. During Units 1 to 8, the teacher is encouraged to foster exchange and discussions among students through the use of interactive games, role-plays, and other interactive instructional methods. The teacher is advised to make sure that both immigrant and non-immigrant youth are able to equally contribute to class activities (equal status) and to frame the tasks as requiring contributions from the whole class (common goals). Furthermore, the teacher is asked to support positive exchange among non-immigrant and immigrant youth (support from authorities) and create group settings in which immigrant and non-immigrant youth are mixed (cooperation). In Unit 9, the focus of the class project changes. During Units 9-13, the class is asked to identify a common, positive, and realistic activity that can be carried out together during a project day. The teacher’s role is to foster a group process that enables an experience of common success. Thus, the teacher helps the students find a cooperative structure and supervises them as they plan and carry out the activity. On the individual level, teachers are trained to recognize and differentiate bullies, victims, and bully-victims by providing them with knowledge regarding the mechanisms of reactive and instrumental aggressive behavior. They are also taught how to conduct structured conversations with these groups of students (Roland & Vaaland, 2006). Several studies have been published relying on data from the ViSC program (e.g., Bardach et al., 2021; Gradinger et al., 2012; Gradiner et al., 2015; Gradinger et al., 2016; Yanagida et al., 2016; Yanagida et al., 2019).

### Participants

Two different samples were used to investigate program effectiveness (pretest and posttest) and program sustainability (posttest and follow-up test). As described in detail in the Design and Procedures section, for practical reasons due to limited resources, data collection for the follow-up test was only possible in a smaller number of schools and classes. Whereas the pretest-posttest sample for investigating program effectiveness comprised 18 schools, the posttest-follow-up sample for investigating program sustainability comprised 6 schools (see Table 1).

#### Pretest—Posttest Sample

In total, 2042 students (1,377 in the intervention group, 665 in the control group) from 18 schools participated in at least one measurement occasion and were thus included in the current study. At pretest, the sample comprised 1639 students (47.6% girls) from 103 classrooms (50 fifth-grade classes, 51 sixth-grade classes and two seventh-grade classes) with a mean age of 11.7 years (*SD* = 0.9, Min = 10, Max = 15). Of the participants. 46.4% were native-born (from Austria), 20.2% from the former Yugoslavia, 14.3% from Turkey, and 19.1% from other countries.

#### Posttest – Follow-up Sample

In total, 659 students (319 in the intervention group, 340 in the control group) from 6 schools participated in at least one measurement occasion and were thus included in the current study. At posttest, the sample comprised 522 students (47.9% girls) from 35 classrooms (18 sixth-grade classes and 17 seventh-grade classes) with a mean age of 12.7 years (*SD* = 0.8, Min = 11, Max = 15). Regarding ethnicity, 43.9% of the students were native-born (from Austria), 20.6% from the former Yugoslavia, 16.1% from Turkey, and 19.4% from other countries.

### Missing Data

Patterns of missing data across the pretest, posttest and follow-up test are shown in Table S1. Differences between participants with complete data and participants with wave nonresponse regarding aggressive behavior and victimization measures at pretest and posttest are displayed in Tables S2 and S3 in the Online Supplement. Readers can find more detailed information on missing data in the pretest – posttest sample and the posttest – follow-up test sample in Online Supplement S1. We also describe the missing data approach (multiple imputation) used for the data analyzed in the current study in Online Supplement S1.

### Measures

All variables were measured at all three waves.

#### Demographic information

Gender, age, and country of birth were assessed with multiple-choice items. Country of birth was measured with the question “In which country were you born?” Students could select their country of origin from a list of countries provided with this item.

#### Aggressive behavior and victimization

With the aim of measuring aggressive behavior and victimization broadly (see also e.g., Ladd et al., 2017), aggressive behavior was measured with three scales capturing different types of aggressive behavior (physical aggression, relational aggression, bullying perpetration), and victimization was measured with three scales capturing different types of victimization (physical victimization, relational victimization, bullying victimization). A list of all items making up each scale is provided in Online Supplement S2. For all scales assessing types of aggressive behavior and victimization, a five-point response scale was used with the following categories: 0 (*not at all*), 1 (*once or twice*), 2 (*two or three* *times a month*), 3 (*once a week*), and 4 (*nearly every day*).

##### Physical aggressive behavior and physical victimization

The peer nomination measure developed by Crick and Grotpeter (1995) was modified into a self-report questionnaire consisting of three items each for physical aggressive behavior and victimization (e.g., “How often have you hit one or more classmates during the last two months?” for physical aggression, and “How often have you been hit by one or more classmates during the last two months?” for physical victimization). Cronbach’s α coefficients for the physical aggressive behavior scale were 0.79/0.79 (pretest/posttest) and 0.79/0.75 (posttest/follow-up test). Cronbach’s α coefficients for the physical victimization scale were 0.74/0.76 (pretest/posttest) and 0.75/0.73 (posttest/follow-up test).

##### Relational aggressive behavior and relational victimization

Five items each for relational aggressive behavior and victimization, respectively, were also adapted from the peer nomination measure originally developed by Crick and Grotpeter (1995). A sample item for relational aggressive behavior is “Some kids leave other kids out on purpose when it’s time to play or do an activity. How often have you done that during the last two months?”, while a sample item for relational victimization is “How often during the last two months have you been excluded from play or another activity by one or more classmates?”. Cronbach’s α coefficients for the relational aggressive behavior scale were 0.83/0.87 (pretest/posttest) and 0.85/0.81 (posttest/follow-up test). Cronbach’s α coefficients for the relational victimization scale were 0.82/0.81 (pretest/posttest) and 0.82/0.78 (posttest/follow-up test).

##### Bullying perpetration and bullying victimization

Self-reported bullying and victimization were each measured with a scale consisting of a global item and three specific items covering different forms of bullying (Strohmeier et al., 2012). The global items for bullying perpetration and bullying victimization, respectively, were “How often have you insulted or hurt other students during the last two months?” and (“How often have others insulted or hurt you in the last two months?”). Cronbach’s α coefficients for the bullying perpetration scale were 0.82/0.83 (pretest/posttest) and 0.80/0.72 (posttest/follow-up test). Cronbach’s α coefficients for the bullying victimization scale were 0.81/0.82 (pretest/posttest) and 0.82/0.79 (posttest/follow-up test).

#### Positive Social Class Climate

We assessed positive social class climate with three items developed by Eder and Mayr (2000): “In our class, all students work together well and help each other”, “In our class it is important for everyone that we all get along well”, and “In our class having a good class community is important to all of us”. These items were answered using a four-point Likert scale, with the response options 0 (*not at all true*), 1 (*somewhat true*), 2 (*true*), and 3 (*certainly true*). Cronbach’s α coefficients for the class climate scale were 0.85/0.86 (pretest/posttest) and 0.90/0.86 (posttest/follow-up test).

#### Ethnic diversity

Ethnic diversity in the class was measured using a formula developed by Simpson (1949, see in Juvonen et al., 2006, p. 394).

1 $$D_c = 1 - \mathop {\sum }\limits_{i = 1}^g p_i^2$$

*D*_*c*_ represents the ethnic diversity of a given class *c*, and _*pi*_ is the proportion of students in the class who belong to ethnic group *i*. The _*pi*_^*2*^ is summed across *g* groups in a class. For our study, the ethnic diversity index was calculated based on the students’ country of origin. Because the sample comprised of more than 40 different countries of origin, seven groups were used to calculate the diversity index: native-born (Austria), Turkey, former Yugoslavia, Eastern Europe, other Western countries, Africa, and Asia. Country of origin (and not self-reported ethnicity) is a more meaningful category in many European countries. It is not common in Austria to classify people according to ethnicity, as in the US or UK (e.g., “Caucasian”, “Latinx”, etc.), as most immigrants are Caucasian, but still speak a different language due to their national origin. The range of this index in the present sample with a maximum of seven groups lies between 0 and 0.86, with higher scores indicating more diversity.

### Measurement models

The adequacy of the measurement models for all multiple-item measures (aggressive behavior, victimization, and positive social class climate) was tested, including measurement invariance testing. All analyses were performed separately for the pretest-posttest sample and the posttest-follow-up sample. In line with our broad conceptualization of aggressive behavior and victimization (see also Ladd et al., 2017), the measurement model for aggressive behavior was based on a second-order factor model with bullying perpetration, physical aggression, and relational aggression as first-order factors, while the measurement model for victimization was based on a second-order factor model with bullying victimization, physical victimization, and relational victimization as first-order factors. In summary, all measurement models yielded a very good model fit for pretest – posttest and posttest – follow-up; strong measurement invariance was supported. Online Supplement S3 and Table S4 provide detailed information on the measurement models, measurement invariance testing, and respective results. Table S5 (program effectiveness) and Table S6 (program sustainability) report correlations between the scales measuring aggressive behavior and victimization.

### Analytic Strategies

Multilevel modeling (Hox et al., 2018) was used to investigate program effectiveness (pretest – posttest) and sustainability (posttest – follow-up test), taking class- and individual-level moderators and the nested data structure into account. From the measurement models for aggressive behavior and victimization (see description of measurement models in the Measures section), factor scores for the second-order factors for aggressive behavior and victimization, respectively, were extracted from the measurement models (see Table S4 in the Online Supplement for all measurement models). The factor scores were then used compute difference scores (*posttest minus pretest* and *follow-up test minus posttest*) and subsequently entered as dependent variables in the main analysis.

A series of models were specified. Given the multi-level modeling approach taken in the current study, a null model was set up to examine the proportion of variance in the dependent variables at the class-level (Model 0). Building upon this first model, four further models were estimated to investigate the four research questions. To address Research Question 1, in Model 1, the predictor *intervention* was additionally included to test program effectiveness and sustainability.

In Model 2 (Research Question 2), covariates at the individual and class-level were additionally included to test program effectiveness and sustainability while controlling for a set of covariates. For positive social class climate, which was modeled at the class-level, factor scores for the class-level factor positive social class climate were extracted from the measurement model (see Table S4 in the Online Supplement). All covariates at the individual level were centered at the group mean, while metric covariates at the class-level were centered at the grand mean. In Model 3, the interaction effects *intervention x positive social class climate* and *intervention x ethnic diversity* were additionally included to test for moderators on the class-level (Research Question 3).

In Model 4, individual-level moderators were additionally considered (Research Question 4). In order to test for individual-level moderators, as a first step, it was necessary to test random slope effects of all covariates on the individual level using deviance tests (see Snijders & Bosker, 2012). Random slopes explain heterogeneity in the slope parameter between classes (i.e., indicate how a link between variables varies between classrooms) and are required for testing cross-level interactions. Cross-level interactions were then used to predict the between-class variability of the slope parameters for the covariates found significant in Step 1 using the predictor *intervention*. The cross-level interaction *intervention x covariate* represents the moderating effect of an individual-level covariate on program effectiveness or sustainability. It should be noted that in all models including interactions, the other main effects must be interpreted as conditional main effects. For example, in a model including effects of the intervention as well as the interaction term between the intervention and a covariate, a significant main effect indicates that there is an intervention effect at average levels of the covariate.

The models were estimated in Mplus 8.6 (Muthén & Muthén, 1998 - 2017) using maximum likelihood estimation with robust standard errors (MLR). All analyses were conducted using two-tailed tests. Significance testing was performed at the α = 0.05 level.

## Results

### Descriptive Statistics

Inspecting the means and standard deviations of aggressive behavior and victimization at pretest, posttest and follow-up test separately for the intervention and control group (see Table 2) as well as the change scores for aggressive behavior and victimization from pretest to posttest and from posttest to follow-up test separately for the intervention and control group (see Table 3) revealed that the control group experienced an increase in aggressive behavior, while the intervention group showed a slight decrease from pretest to posttest. For victimization, there was a decrease in both the control and intervention groups from pretest to posttest, but the decrease was much stronger in the intervention group. Between posttest and follow-up test, aggressive behavior and victimization decreased in both the control and intervention group, although the decrease in the intervention group was much stronger for both outcome variables.

**Table 2:** Descriptive statistics for aggressive behavior and victimization: means and standard deviations by intervention and control group

	Pretest		Posttest		Follow-up test	
	Intervention	Control	Intervention	Control	Intervention	Control
	*n* = 1181	*n* = 436	*n* = 934	*n* = 588	*n* = 256	*n* = 242
Aggressive behavior	0.44 (0.51)	0.40 (0.45)	0.46 (0.59)	0.47 (0.58)	0.33 (0.49)	0.44 (0.58)
Victimization	0.64 (0.71)	0.58 (0.62)	0.47 (0.61)	0.54 (0.68)	0.44 (0.59)	0.49 (0.69)

*Note*. Scale mean scores were used for these analyses

**Table 3:** Means and standard deviations of aggressive behavior change and victimization change

	Posttest – Pretest (*n* = 2042)		Follow-up test - Posttest (*n* = 659)	
	ΔAggressive behavior	ΔVictimization	ΔAggressive behavior	ΔVictimization
Control				
*M*	0.03	−0.02	−0.03	−0.07
*SD*	0.93	1.08	0.64	0.99
Intervention				
*M*	−0.01	−0.34	−0.29	−0.19
*SD*	0.97	1.06	0.41	0.74

*Note*. Factor scores were used for these analyses

### Intraclass Correlation

To examine the proportion of variance in the dependent variables at the class-level, the intraclass correlations (ICC) for the change scores in aggressive behavior and victimization from pretest to posttest and post-test to follow-up were computed separately based on the null model. Results indicated that between 2.1% and 7.8% of the variance was between classes (see Table S7 in the supplemental material). Despite the rather low ICCs, multilevel analysis is an appropriate analytic strategy for multilevel data and needed to account for design effects, examine random slope effects and investigate moderator effects at the individual and class-level (e.g., Snijders & Bosker, 2012).

### Program Effectiveness

The aggression prevention program should work as intended, in that aggressive behavior and victimization would show a more advantageous trajectory of change from pretest to posttest in the intervention group than in the control group (Hypothesis 1a). The results revealed that the control group’s aggressive behavior and victimization did not change between pre- and posttest, as indicated by statistically non-significant intercepts for the change in aggressive behavior (*b* = 0.031, *p* = 0.451) and in victimization (*b* = −0.021, *p* = 0.674). However, *intervention* was a statistically significant predictor for the change in victimization (*b* = −0.316, *p* < 0.001), meaning that a decrease in victimization between pre- and posttest in the intervention group was found. No statistically significant effect of the predictor *intervention* emerged for the change in aggressive behavior (*b* = −0.037, *p* = 0.502). These results indicate program effectiveness for victimization, but not for aggressive behavior.

The results for program effectiveness should not be substantially affected by the inclusion of covariates at the class-level and individual-level (Hypothesis 2a). As can be seen in Table 4 (Model 2) the pattern of results remained the same as in the analyses without covariates, demonstrating the effectiveness of the intervention regarding victimization, but not aggressive behavior. Moreover, for changes in aggressive behavior, significant effects of the covariates gender (stronger increase for boys), and aggressive behavior and victimization at pretest (the higher one’s aggressive behavior and victimization at pretest, the stronger the decrease in aggressive behavior) were found.

**Table 4:** Multilevel modeling results: effectiveness (posttest – pretest) for aggressive behavior and victimization

	Posttest - Pretest											
	Model 2				Model 3				Model 4			
	ΔAggressive behavior		ΔVictimization		ΔAggressive behavior		ΔVictimization		ΔAggressive behavior		ΔVictimization	
Coefficient	Est.	SE	Est.	SE	Est.	SE	Est.	SE	Est.	SE	Est.	SE
Level 1 – Individual												
Aggressive behavior at pretest	−**0.42**	0.05	−0.09	0.05	−**0.42**	0.05	−0.09	0.05	−0.01	0.09	−0.08	0.12
Victimization at pretest	−**0.08**	0.04	−**0.44**	0.05	−**0.08**	0.04	−**0.44**	0.05	−**0.45**	0.08	−**0.26**	0.11
Age at pretest	0.03	0.03	−0.01	0.04	0.03	0.03	−0.01	0.04	0.02	0.03	−0.07	0.06
Gender (0 = female, 1 = male)	**0.14**	0.05	0.02	0.05	**0.14**	0.05	0.02	0.05	0.12	0.09	0.02	0.11
Level 2 – Class												
Intercept	0.02	0.05	−0.04	0.06	0.03	0.04	−0.01	0.05	0.03	0.04	−0.01	0.05
Intervention (0 = control, 1 = intervention)	−0.01	0.06	−0.29	0.08	−0.02	0.05	−**0.31**	0.07	−0.02	0.05	−**0.31**	0.07
Class climate at pretest	**0.21**	0.09	0.29	0.10	−0.17	0.16	−0.32	0.18	−0.17	0.16	−0.32	0.18
Ethnic diversity at pretest	0.02	0.12	−0.14	0.14	**0.51**	0.25	0.45	0.28	**0.51**	0.25	0.44	0.28
Intervention x class climate at pretest					**0.52**	0.20	**0.85**	0.22	**0.51**	0.20	**0.85**	0.22
Intervention x ethnic diversity					−**0.62**	0.29	−**0.65**	0.33	−**0.63**	0.29	−**0.64**	0.33
Intervention x aggressive behavior at pretest									−**0.52**	0.11	−0.04	0.13
Intervention x victimization at pretest									**0.45**	0.45	−**0.23**	0.12
Intervention x gender									0.02	0.10	0.00	0.12
Variance components												
Level 1 – individual	0.69		0.82		0.69		0.82		0.66		0.77	
Level 2 – class	0.03		0.04		0.02		0.03		0.02		0.03	
Slope aggressive behavior at pretest									0.01		0.00	
Slope victimization at pretest									0.00		0.01	
Slope gender									0.01		0.02	
R-Squared												
Level 1 – individual	0.22		0.25		0.22		0.25					
Level 2 – class	0.25		0.44		0.36		0.63					
Model summary												
Deviance	9135.60				9114.65				8846.89			
AIC	9179.60				9166.65				8926.89			
BIC	9303.28				9312.82				9151.76			

*Note*. Unstandardized coefficients

Gender is coded as 0 = girls and 1 = boys; Intervention is coded as 0 = control group and 1 = intervention group

Statistically significant coefficients at α = 0.05 are shown in boldface

### Program Sustainability

The more advantageous trajectory of change over time in the intervention group than in the control group should also be found for the period from posttest to follow-up test (Hypothesis 1b). The results for program sustainability (Model 1) showed no change in aggressive behavior and victimization in the control group, as indicated by the not statistically significant *intercepts* for the change in aggressive behavior (*b* = −0.030, *p* = 0.495) and in victimization (*b* = −0.084, *p* = 0.348). However, the predictor *intervention* was statistically significant for the change in aggressive behavior (*b* = −0.260, *p* < 0.001), indicating a reduction in aggressive behavior after the posttest in the intervention group (sleeper effect). For the change of victimization, the predictor *intervention* was statistically non-significant (*b* = −0.108, *p* = 0.357), indicating a similar trend in both groups after the posttest. This means that program’s effects on victimization were sustained.

The program sustainability effects were assumed to remain robust when controlling for a set of individual-level and class-level covariates (Hypothesis 2b). As can be seen in Table 5 (Model 2, Table 5), these results revealed a decrease in aggressive behavior in the intervention group after the posttest (sleeper effect) and sustainability of the intervention effect on victimization. For changes in aggressive behavior, the covariates aggressive behavior and victimization at posttest were statistically significant. These results indicate that the higher one’s aggressive behavior at posttest, the stronger the decrease in aggressive behavior from posttest to follow-up, while the higher the victimization at posttest, the stronger the increase in aggressive behavior from posttest to follow-up. Regarding changes in victimization, the covariates aggressive behavior at posttest and victimization at posttest were statistically significant: The higher the aggressive behavior at posttest, the stronger the increase in victimization from posttest to follow-up, while the higher the victimization at posttest, the stronger the decrease in victimization from posttest to follow-up (see Table 5).

**Table 5:** Multilevel modeling results: sustainability (follow-up test - posttest) for aggressive behavior and victimization

	Follow-up test - Posttest											
	Model 2				Model 3				Model 4			
	ΔAggressive behavior		ΔVictimization		ΔAggressive behavior		ΔVictimization		ΔAggressive behavior		ΔVictimization	
Coefficient	Est.	SE	Est.	SE	Est.	SE	Est.	SE	Est.	SE	Est.	SE
Level 1 – Individual												
Aggressive behavior at posttest	−**0.37**	0.06	**0.17**	0.08	−**0.37**	0.066	**0.17**	0.08	0.01	0.06	**0.17**	0.08
Victimization at posttest	**0.06**	0.03	−**0.37**	0.04	**0.06**	0.03	−**0.37**	0.04	0.05	0.03	−**0.37**	0.04
Age at posttest	0.00	0.03	0.01	0.05	0.00	0.03	0.01	0.05	−0.01	0.03	0.01	0.05
Gender (0 = girls, 1 = boys)	0.00	0.04	−0.03	0.07	0.00	0.04	−0.03	0.07	−0.01	0.04	−0.03	0.07
Level 2 – Class												
Intercept	0.02	0.05	−0.03	0.08	0.01	0.06	−0.08	0.08	0.01	0.06	−0.08	0.08
Intervention (0 = control, 1 = intervention)	−**0.35**	0.08	−0.23	0.13	−**0.32**	0.08	−0.08	0.12	−**0.32**	0.07	−0.08	0.12
Class climate at posttest	0.19	0.12	0.43	0.22	0.21	0.16	**0.49**	0.23	0.22	0.16	**0.50**	0.23
Ethnic diversity at posttest	−0.15	0.18	0.44	0.52	0.16	0.37	**1.70**	0.77	0.16	0.36	**1.69**	0.78
Intervention x class climate at posttest					−0.21	0.40	−**0.80**	0.29	−0.22	0.18	−**0.80**	0.29
Intervention x ethnic diversity					−0.56	0.18	−**2.28**	0.83	−0.56	0.39	−**2.27**	0.83
Intervention x aggressive behavior at posttest									**0.22**	0.06		
Variance components												
Level 1 – individual	0.22		0.60		0.22		0.60		0.20		0.60	
Level 2 – class	0.01		0.05		0.01		0.02		0.01		0.02	
Slope aggressive behavior at posttest											0.00	
R-Squared												
Level 1 – individual	0.21			0.17	.21		0.17					
Level 2 – class	0.71			0.34	.80		0.75					
Model summary												
Deviance	2074.63				2057.67				2023.03			
AIC	2118.63				2109.67				2079.03			
BIC	2217.42				2226.43				2204.77			

*Note*. Unstandardized coefficients

Gender is coded as 0 = girls and 1 = boys; Intervention is coded as 0 = control group and 1 = intervention group

Statistically significant coefficients at α = 0.05 are shown in boldface

### Class-level Moderators of Effectiveness

Higher levels of a more positive social class climate should strengthen program effectiveness (Hypothesis 3a). No hypothesis was specified for ethnic diversity as a potential mediator, and exploratory analyses were conducted. In the model including the interactions (Model 3, Table 4), for changes in aggressive behavior, *ethnic diversity at pretest* was statistically significant (*b* = 0.506, *p* = 0.041). This indicates that the higher the ethnic diversity in the control group, the stronger the increase in aggressive behavior in the control group, controlling for all other covariates in the model. Moreover, a statistically significant interaction effect for *intervention x ethnic diversity* was obtained (*b* = −0.622, *p* = 0.030). More specifically, the *intervention* effect given average levels of ethnic diversity and positive class climate (conditional main effect) was statistically non-significant (*b* = −0.021, *p* = 0.703); however, the intervention effect increased with increasing ethnic diversity. This means that there was no relationship between ethnic diversity and changes in aggressive behavior in the intervention group, while high ethnic diversity in the control group was associated with an increase in aggressive behavior. The results further indicated that *positive social class climate at pretest* was not related to the change in aggressive behavior in the control group (*b* = −0.171, *p* = 0.290). The statistically significant interaction effect *intervention x class climate* (*b* = 0.515, *p* = 0.011) revealed that the intervention effect decreased with increasing positive social class climate. This means that a more positive social class climate in the intervention group was associated with a stronger increase in aggressive behavior compared to the control group.

Regarding changes in victimization, the predictor *intervention* (*b* = −0.308, *p* < 0.001), the interaction term *intervention x positive social class climate at pretest* (*b* = 0.845, *p* < 0.001) and the interaction term *intervention x ethnic diversity at pretest* (*b* = −0.645, *p* = 0.047) were statistically significant. The effect for the predictor *intervention* can be interpreted as evidence of program effectiveness for victimization at average levels of positive social class climate and ethnic diversity (conditional main effect). However, the statistically significant interaction effect *intervention x positive social class climate at pretest* indicated that the more positive the social class climate in the intervention group, the stronger the increase in victimization in the intervention group as compared to the control group. Moreover, the statistically significant interaction effect *intervention x ethnic diversity at pretest* revealed that the higher the ethnic diversity in the intervention group, the stronger the decrease in victimization in the intervention group, as compared to the control group.

### Class-level Moderators of Sustainability

A more positive social class climate should increase the intervention effects (Hypothesis 3b), whereas no concrete hypothesis was outlined for ethnic diversity. For changes in aggressive behavior, the predictor *intervention* (*b* = −0.318, *p* < 0.001) was statistically significant in the model including the interactions (see Table 5, Model 3). This result indicated an intervention effect at average levels of positive social class climate and ethnic diversity (conditional main effect). Regarding victimization, the conditional main effects *positive social class climate at posttest* (*b* = 0.493, *p* = 0.029) and *ethnic diversity* (*b* = 1.700, *p* = 0.028) as well as interaction effects *intervention x positive social class climate at posttest* (*b* = −0.800, *p* = 0.005) and *intervention x ethnic diversity* (*b* = −2.283, *p* = 0.006) were statistically significant. These findings showed that (a) the higher the positive social class climate and ethnic diversity, the stronger the increase in victimization in the control group, while (b) the higher the positive social class climate and ethnic diversity, the stronger the decrease in victimization in the intervention group compared to the control group (i.e., higher intervention effects).

### Individual-level Moderators of Effectiveness

Stronger intervention effects on changes in aggressive behavior should surface in the presence of higher baseline levels of aggressive behavior at pretest, while stronger effects on victimization should surface in the presence of higher baseline levels of victimization at pretest (Hypothesis 4a). Moreover, stronger intervention effects on changes in victimization (aggressive behavior) should occur in the presence of higher baseline levels of aggressive behavior (victimization) at pretest (Hypothesis 4c). Testing the variability of the slope parameters (i.e., random slopes) between classes for all individual-level covariates using a series of deviance tests revealed that for changes in aggressive behavior, there were random slope effects for *aggressive behavior at pretest* (χ^2^ (1) = 9.93, *p* = 0.002), *victimization at pretest* (χ^2^(1) = 23.52, *p* < 0.001), and *gender* (χ^2^(1) = 4.56, *p* = 0.033). For changes in victimization, there were random slope effects for *aggressive behavior at pretest* (χ^2^(1) = 11.43, *p* < 0.001), *victimization at pretest* (χ^2^(1) = 64.23, *p* < 0.001), and *gender* (χ^2^(1) = 5.04, *p* = 0.025) (see Table 6). The predictor *intervention* was then used to predict the variability of the slope parameters for the covariates found to be significant between classes (i.e., cross-level interactions were estimated). For changes in aggressive behavior (see Table 4, Model 4), the cross-level interactions *intervention x aggressive behavior at pretest* (*b* = −0.522, *p* < 0.001) and *intervention x victimization at pretest* (*b* = 0.454, *p* < 0.001) were statistically significant. This indicates that the higher the aggressive behavior at pretest, the stronger the decrease in aggressive behavior in the intervention group compared to the control group (i.e., higher intervention effects) and the higher the victimization at pretest, the smaller the decrease in aggressive behavior in the intervention group compared to the control group (i.e., smaller intervention effects). Regarding changes in victimization, the cross-level interaction *intervention x victimization at pretest* (*b* = −0.233, *p* = 0.045) was statistically significant, indicating the higher the victimization at pretest, the stronger the decrease in victimization in the intervention group compared to the control group (i.e., higher intervention effects). All other cross-level interactions for changes in aggressive behavior and victimization were statistically non-significant (see Table 4, Model 4).

**Table 6:** Deviance test of random slopes

	Posttest – Pretest				Follow-up test - Posttest			
	ΔAggressive Behavior		ΔVictimization		ΔAggressive Behavior		ΔVictimization	
Variance components	*D*_0_ – *D*_1_	*p*	*D*_0_ – *D*_1_	*p*	*D*_0_ – *D*_1_	*p*	*D*_0_ – *D*_1_	*p*
Slope aggressive behavior	**9.93**	0.002	**11.43**	<0.001	**16.61**	<0.001	−0.15	1.000
Slope victimization	**23.52**	<0.001	**64.23**	<0.001	0.30	0.581	1.58	0.209
Slope gender	**4.56**	0.033	**5.04**	0.025	0.21	0.645	−0.19	1.000

*Note*. Statistically significant coefficients at α = 0.05 are shown in boldface

### Individual-level Moderators of Sustainability

Stronger intervention effects on changes in aggressive behavior were expected in the presence of higher baseline levels of aggressive behavior at posttest and stronger effects on victimization in the presence of higher baseline levels of victimization at posttest (Hypothesis 4b). Moreover, stronger intervention effects on changes in victimization (aggressive behavior) should occur in the presence of higher baseline levels of aggressive behavior (victimization) at posttest (Hypothesis 4d). For changes in aggressive behavior, a random slope effect for *aggressive behavior at posttest* (χ^2^(1) = 16.61, *p* < 0.001) was found. For changes in victimization, no statistically significant random slope effects were obtained (see Table 6 for all deviance test results). The cross-level interaction *intervention x aggressive behavior at posttest* (*b* = 0.219, *p* < 0.001) was statistically significant for changes in aggressive behavior (see Table 5, Model 4). This indicates that the higher the aggressive behavior at posttest, the smaller the decrease in aggressive behavior in the intervention group as compared to the control group (i.e., smaller intervention effect), controlling for all other covariates.

### Alternative Model Specifications

Further analyses with alternative model specifications were conducted. Specifically, the models were re-run using grand-mean instead of group-mean centering for the individual-level predictors. The results remained largely the same, with four major exceptions: First, in the alternative models with grand-mean centered individual-level predictors, a positive social class climate was significantly related to more adaptive changes in aggressive behavior and victimization in the control group between pretest and posttest. The results in the main analyses relying on group-mean centered individual-level predictors yielded a non-significant effect for the control group. Second, the alternative models for program sustainability showed not only decreases in victimization but also in aggressive behavior after the posttest for intervention classes with a more positive class climate. In the main analyses, significant effects were restricted to victimization. Third, in the alternative models, ethnic diversity moderated only program sustainability, not effectiveness. Moderating effects for both effectiveness and sustainability were found in the main analyses. Fourth, in the alternative models, moderating effects of baseline levels of both aggressive behavior and victimization at posttest were obtained. In the main analyses, significant moderating effects were only documented for aggressive behavior. All results for the alternative model specifications can be consulted in Tables S8 (program effectiveness) and S9 (program sustainability) in the Online Supplement, and a detailed description of the findings can be found in Online Supplement S4. These results are reported to provide additional, more comprehensive information; however, this study’s findings are interpreted and discussed based on the results from the main analyses employing group-mean centering for the individual-level predictors, as this is the more appropriate approach (e.g., Enders and Tofighi, 2007).

## Discussion

Aggressive behavior reaches a peak in preadolescence (Inchley et al., 2016). In order to better understand the nature and effects of aggression interventions designed to counteract preadolescents’ engagement in aggressive behavior and victimization, researchers need to start disentangling the complex processes leading to differentiated and potentially contrasting outcomes (e.g., Juvonen et al., 2016). The present study advanced current knowledge of the functioning of the ViSC aggression prevention program by testing whether individual student characteristics (baseline levels of aggressive behavior and victimization, gender) and classroom characteristics (positive social class climate, ethnic diversity) moderate the program’s effectiveness and sustainability.

Prior to testing moderator effects, the program’s effectiveness and sustainability was examined, with and without controlling for a set of covariates in order to ensure that any intervention effects were not caused by co-varying individual- and class-level characteristics. To summarize, the results of the analyses with and without covariates demonstrated the intervention’s effectiveness regarding victimization, but not aggressive behavior. Next, program sustainability was investigated (i.e., changes between posttest and follow-up test) to gain clarity about potential intervention effects six months after the conclusion of the intervention. Most interventions aim for sustained change in the desired direction. Of particular relevance for practice, it must be ruled out that interventions backfire once the program ends (generate changes in the direction opposite of what was intended, see e.g., Johander et al., 2020). Program sustainability in terms of changes between posttest and follow-up test were apparent for both aggressive behavior and victimization in the models with and without covariates. Importantly, aggressive behavior was not significantly affected by the intervention during the intervention period; however, reduced aggressive behavior after the intervention in the intervention group was found (sleeper effect). Hence, more time seems to be needed for the program’s effects on aggressive behavior to materialize. For example, it may be the case that beneficial classroom practices, improved interactions, and further systemic changes are not fully established during the intervention and may instead manifest after the intervention, thus resulting in gradually increasing intervention effects for aggressive behavior over time. For victimization, positive intervention effects were sustained from posttest to follow-up. Overall, the results regarding program sustainability highlight the need for continued research after aggression interventions end. For practitioners, the increased and self-reinforcing effects obtained in the analyses for program sustainability inspire further confidence in the usefulness of the investigated aggression prevention program.

In the analyses of class-level characteristics as moderators of program effectiveness, positive social class climate was not significantly related to changes in aggressive behavior and victimization in the control group. Hence, a more positive social class climate did not engender more adaptive changes in both outcomes, contradicting basic tenets of socio-ecological theory stating that a positive social environment is critical to reducing aggression (e.g., Espelage, 2014). In the intervention group, a positive social class climate did serve as a mechanism of change for program effectiveness regarding victimization. However, the direction of effects was unexpected as more positive class climate at pretest was conducive of a smaller intervention effect at posttest. The reasons for this finding are difficult to establish. A potential explanation builds on the healthy context paradox. The healthy context paradox was originally developed to describe the unexpected pattern that victims’ psychological adjustment worsens as the overall level of victimization in a classroom or school declines (e.g., Garandeau & Salmivalli, 2019; Yun & Juvonen, 2020). It is cautiously proposed that a similar healthy context paradox may underlie this study’s finding, as in socially well-functioning classes (high positive social class climate), students may be more sensitive to victimization, feel victimized more easily and experience more distress, particularly if they learn about victimization and become more aware of their own victimization experiences as part of the intervention. For program effectiveness, the effects on changes in aggressive behavior mirrored those for victimization. Whereas no significant link between positive social class climate and changes in aggressive behavior was found in the control group, a more positive social class climate in the intervention group predicted increases in aggressive behavior. The same health context paradox explanation outlined for victimization may hold for these finding as well, and more research is clearly needed to further probe this possibility.

The inclusion of the follow-up test data demonstrated a moderating effect on sustainability for victimization, in that a more positive class climate at posttest magnified the effects of the intervention on victimization. As such, the moderating effect of positive social class climate on sustainability contrasted the findings of the moderator analyses for program effectiveness (see paragraph above), with a larger intervention effect (for program sustainability) as opposed to an increase in victimization (for program effectiveness) at higher levels of positive social class climate. Furthermore, surprisingly, a more positive class climate at posttest in the control group was indicative of subsequent increases in victimization, thus contradicting the large body of research linking class and school climate to lower levels of victimization.

The second classroom characteristic, ethnic diversity, significantly moderated program effectiveness and sustainability. For program effectiveness, ethnic diversity did not relate to changes in aggressive behavior in the intervention group. However, higher ethnic diversity in the control group was associated with an increase in aggressive behavior. Significant moderation effects for program effectiveness were also found for victimization, in that stronger decreases in victimization in the intervention group emerged in the presence of higher ethnic diversity. Ethnic diversity was not significantly linked to changes in victimization in the control group. The findings for program sustainability were even more illuminating. Whereas higher ethnic diversity was related to a stronger increase in victimization in the control group after posttest, the opposite pattern for the intervention group was obtained. In the intervention group, higher ethnic diversity forecasted a stronger decrease in victimization following the intervention period. These results extend prior correlation-based research linking ethnic diversity to aggression, which has sometimes produced contrasting findings as to whether ethnic diversity should best be conceived of as a risk or protective factor (e.g., Jansen et al., 2016; Juvonen et al., 2006). Our study’s findings, particularly those for program sustainability, reveal ethnic diversity to be a double-edged sword. In the intervention group, it served as a protective factor, probably because the program included elements fostering social and intercultural competencies, which prompted students to value ethnic diversity and see it as an opportunity for exchange and personal growth rather than a threat (e.g., Schachner, 2019). On the other hand, in the control group, ethnic diversity predicted increases in victimization, providing support for the risk-factor perspective (see also e.g., Jansen et al., 2016). Adaptive approaches to dealing with ethnic and cultural diversity, which may go hand in hand with reduced aggression, might not evolve spontaneously and should be explicitly fostered and reinforced at school, for example by implementing interventions like the one presented here.

Finally, as individual differences warrant systematic scholarly attention in order to improve the effectiveness of treatments for aggression (Hendriks et al., 2018) this study investigated three individual-level moderators. Gender did not turn out to be a significant moderator of either program effectiveness or program sustainability. It can therefore be concluded that the program yields the same effects for boys and girls. In accordance with risk x intervention interactions found in previous research (Juvonen et al., 2016), higher aggressive behavior at pretest yielded stronger intervention effects in the sense of decreased aggressive behavior in the intervention group. Risk x intervention interactions were furthermore observed for victimization, with larger intervention effects on victimization at higher levels of pretest victimization. At the same time, however, the results indicated smaller intervention effects on aggressive behavior at higher pretest levels of victimization. It could be that in the present sample, students tended not to simultaneously both experience victimization and engage in aggressive behavior. Therefore, the intervention may have not been able to generate meaningful changes in aggressive behavior in the presence of high levels of victimization. In the analyses for sustainability, smaller intervention effects on changes in aggressive behavior emerged among those scoring higher on aggressive behavior at posttest, indicating a “reverse risk x intervention effect”. This could indicate that students with higher levels of aggression would have needed further structured activities and support, as had been provided in the intervention period, in order to continue counteracting their aggressive tendencies.

The following study limitations have to be kept in mind when interpreting the findings. First, the aggressive behavior and victimization measures were based on self-reports. Hence, future studies following up on our work should consider a broader palette of measures and data sources (e.g., observations, parent, peer and teacher ratings) to shed light on the functioning of aggression interventions from different angles. Second, the inclusion of follow-up test data represents a considerable strength of our study; however, these analyses only included a sub-sample of the original sample used to estimate program effectiveness, and it would have been desirable to gather follow-up data in the entire sample. Third, the analyses carried out in the present work make it possible to tackle a number of relevant questions related to moderators of program effectiveness and sustainability. Nonetheless, future studies could delve even deeper into the complex mechanisms of change, for example by estimating mediated moderation models or conducting person-centered investigations, which allow for examining complex configurations of aggression (e.g., students who both engage in aggressive behavior and feel victimized). Fourth, some of the explanations for our findings remain speculative, as the variables that would have been necessary to test these assumptions were not available. For instance, it is not known whether the intervention actually promoted more positive approaches to ethnic and cultural diversity (e.g., Oczlon et al., 2021; Schachner et al., 2019). This leaves much room for further work building on this study, which can put these suggestions to the empirical test.

## Conclusion

Although reducing aggression at school is a major concern for educators, parents, and legislators, selecting evidence-based prevention programs remains a challenge. This issue is further complicated by the fact that the same program may work differently in different national, class or school contexts and for different students. In light of the limited knowledge on moderators of aggression prevention programs, more research is needed to guide practice and advance research. Therefore, focusing on preadolescents, an age group in which aggression represents a widespread problem behavior, the present study tested whether individual student and classroom characteristics moderate the effectiveness and sustainability of a socio-ecological aggression prevention program (ViSC program). The ViSC program was able to reduce victimization immediately after program implementation, and these effects were sustainable. Effects on changes in aggressive behavior could only be detected at follow-up, indicating a sleeper effect. The key finding of the study, however, is that the ViSC program was particularly beneficial for multicultural classes. Given the inconsistent prior findings on ethnic diversity and the lack of research on ethnic diversity as a program moderator, this study makes an important contribution by showing that more adaptive changes in preadolescents’ aggressive behavior and victimization were observed in classes with higher ethnic diversity. Further moderating effects were either partly in line with previous findings (e.g., risk x intervention effects for baseline levels of aggressive behavior and victimization, lack of moderating effects for gender). Other findings and their interpretation expand current thinking and theories; for example, healthy context perspectives were applied to explain the unexpected negative effect of a positive social class climate. Overall, the present work contributes to the construction of a more consolidated body of evidence on the functioning of aggression prevention programs in preadolescence.

## Supplementary Information

Supplementary Information

Supplementary Information

## Footnotes

1 The present study focuses on “traditional” face-to-face aggression; however, it should be acknowledged that aggression can also occur through the use of electronic communication technologies (cyber-aggression or cyber-bullying, e.g., Zych et al., 2019).
