# Supplementary material for: Understanding for Which Students and Classes a Socio-Ecological Aggression Prevention Program Works Best: Testing Individual Student and Class Level Moderators
Source: J Youth Adolesc. 2021 Dec 18;51(2):225–43. doi: 10.1007/s10964-021-01553-6 (PMC8828596; doi:10.1007/s10964-021-01553-6)
Supplement: Supplementary file 1 — Supplementary Information [file 10964_2021_1553_MOESM1_ESM.docx]

**Online Supplement S1: Missing Data**

**Pretest – Posttest Sample**. In the pretest – posttest data, 21.1% of data were missing as a result of two main missing data patterns: students who participated at pretest only (*n* = 515) and students who participated at posttest only (*n* = 403). The percentage of missing values in each of the 56 study variables varied between 0.0% and 28.6%. Analyses of wave nonresponse revealed that participants who missed the posttest had higher levels of aggressive behavior (*d* = 0.12) and victimization at pretest (*d* = 0.12) than participants with complete data (see Table S3 in the Online Supplement). These variables were included in the imputation model for the multiple imputation process.

**Posttest – Follow-up test Sample**. In the posttest – follow-up test data, 20.7% of data were missing as a result of two main missing data patterns: students who participated at posttest only (*n* = 160) and students who participated at follow-up only (*n* = 62). The percentage of missing values for each of the 56 study variables varied between 0.0% and 24.6%. Analyses of wave nonresponse showed no differences between participants with complete data and participants missing the follow-up test (see Table S4 in the supplemental material).

**Multiple imputation**. Multiple imputation (Rubin, 1987) under the missing at random (MAR) assumption was used to deal with missing data. Incomplete variables were imputed under fully conditional specification (van Buuren, Brand, Groothuis-Oudshoorn & Rubin, 2006) based on an inclusive analysis strategy incorporating all variables used in the analyses as well as numerous auxiliary variables into the missing data procedure (Collins, Schafer, & Kam, 2001). In order to account for the hierarchical data structure, 34 scale cluster means and item cluster means were included in the imputation model (Graham, 2012). Each variable was imputed with the predictive mean matching algorithm (van Buuren, 2012) using Tukey’s tricube weighting function (Harrell, 2006). A total of 50 imputed data sets were extracted during the imputation process. In order to preserve interactions between the grouping variable and other variables, the imputation process was conducted for the intervention and control group separately (Little & Rubin, 2002). Calculations were done in R (R Core Team, 2014) using the mice package (van Buuren & Groothuis-Oudshoorn, 2011) and miceadds package (Robitzsch, 2014). Note that methodologists currently regard multiple imputation as a state-of-the-art technique with a strong theoretical foundation, less restrictive assumptions, and the potential for bias reduction and greater power (Schafer & Graham, 2002). For more detailed information about the imputation process, see Yanagida et al. (2019).

**References Cited Here and Not in the Main Manuscript:**

Collins, L. M., Schafer, J. L., & Kam, C.-M. (2001). A comparison of inclusive and restrictive strategies in missing data procedures. *Psychological Methods*, *6*, 330-351. doi: 10.1037/1082-989X.6.4.330

Graham, J. W. (2012). *Missing data. Analysis and design*. New York, NY: Springer.

Little, R. J. A., & Rubin, D. B. (2002). *Statistical analysis with missing data*. Wiley.

Harrell, F. H. (2006). *Regression Modeling Strategies: With Applications to Linear Models, Logistic Regression, and Survival Analysis*. Springer.

Graham, J. W. (2012). *Missing data. Analysis and design*. New York, NY: Springer.

van Buuren, S. (2012). Flexible imputation of missing data. Chapman & Hall/CRC Press.

van Buuren, S., Brand, J. P. L., Groothuis-Oudshoorn C. G. M., & Rubin, D. B. (2006) Fully conditional specification in multivariate imputation. *Journal of Statistical Computation and Simulation*, *76*, 1049–1064. doi: 10.1080/10629360600810434

van Buuren, S. & Groothuis-Oudshoorn, K. (2011). mice: Multivariate imputation by chained equations in R. *Journal of Statistical Software*, *45*, 1-67.

R Core Team (2014). *R: A language and environment for statistical computing*. R Foundation for Statistical Computing, Vienna, Austria. URL http://www.R-project.org/.

Robitzsch, A. (2014). *miceadds: Some additional multiple imputation functions, especially for mice*. R package version 0.12-9. <http://CRAN.R-project.org/package=miceadds>

Rubin, D. B. (1987) *Multiple imputation for nonresponse in surveys.* Wiley & Sons.

Schafer, J. L., & Graham, J. W. (2002). Missing data: Our view of the state of the art. *Psychological Methods*, *7*, 147-177. doi: 10.1037/1082-989X.7.2.147

**Online Supplement S2: Items of the Scales Used to Assess Aggressive Behavior and Victimization**

Aggressive behavior was assessed with the three scales bullying perpetration, physical aggression, and relational aggression. Victimization was assessed with the three scales bullying victimization, physical victimization, and relational victimization.

**Bullying Perpetration**

1. How often have you insulted or hurt other students during the last two months? (global)
2. How often have you insulted or hurt other students by verbally harassing them during the last two months? (verbal)
3. How often have you insulted or hurt other students by socially excluding them during the last two months? (exclusion)
4. How often have you insulted or hurt other students by physically harassing them during the last two months? (physical)

**Bullying Victimization**

1. How often have other students insulted or hurt you during the last two months? (global)
2. How often have other students insulted or hurt you by verbally harassing you during the last two months? (verbal)
3. How often have other students insulted or hurt you by socially excluding you during the last two months? (exclusion)
4. How often have other students insulted or hurt you by physically harassing you during the last two months? (physical)

**Physical Aggression**

1. How often have you hit one or more classmates during the last two months? (hit)
2. How often have you shoved or pushed one or more classmates during the last two months?
3. How often have you kicked one or more classmates or pulled their hair during the last two months?

**Physical Victimization**

1. How often have you been hit by one or more classmates during the last two months?
2. How often have you been shoved around or pushed by one or more classmates during the last two months?
3. How often have you been kicked or had your hair pulled by one or more classmates during the last two months?

**Relational Aggression**

1. How often during the last two months have you excluded one or more classmates from play or another activity?
2. How often during the last two months did you when you were angry about one or more classmates get back at them by not wanting to be together with them anymore?
3. How often during the last two months have you told lies about one or more classmates to make others not like them anymore?
4. How often during the last two months have you told one or more classmates you won’t like them anymore unless they do what you want?
5. How often during the last two months have you kept others from liking one or more classmates by telling mean things about them?

**Relational Victimization**

1. How often during the last two months have you been excluded from play or another activity by one or more classmates?
2. How often during the last two months did one or more classmates who were angry about you got back at you by not wanting to be together with you anymore?
3. How often during the last two months have one or more classmates told lies about you to make others not like you anymore?
4. How often during the last two months have one or more classmates told you that they won’t like you anymore unless you do what they want?
5. How often during the last two months have one or more classmates kept others from liking you by telling mean things about you?

**Online Supplement S3: Measurement Models**

The measurement models for aggressive behavior and victimization were each based on a factor model with ordered-categorical indicators (see Bovaird & Koziol, 2012), because items measuring the frequency of incidents are not continuous. Furthermore, the highly positive-skewed nature of the item response distribution makes a statistical approach based on normal theory inappropriate (Muthén & Kaplan, 1985). For these scales, model specification and identification were based on Millsap and Yun-Tein (2004) using theta parameterization and a robust weighted least squares estimator (WLSMV). The measurement model for class climate relied on a factor model with continuous indicators, given that the items were answered on a four-point Likert scale. Multilevel confirmatory factor analysis was used to model perceptions of class climate on the individual and class level.

**Aggressive behavior and victimization*.*** The measurement models for aggressive behavior and victimization were based on a second-order factor model with bullying perpetration/victimization, physical aggression/victimization, and relational aggression/victimization as first-order factors. The confirmatory factor analysis (CFA) for the measurement models assuming strong longitudinal and between-group (control vs. intervention) invariance yielded very good model fit for pretest – posttest and posttest – follow-up test (see Table S4 in the Online Supplement). These results indicate sound measurement properties for both scales.

**Class climate.** The measurement model for class climate consisted of a one-factor multilevel model comprising three continuous indicators. Multilevel CFA for the measurement model assuming strong longitudinal, between-group (control vs. intervention), and within- and between-factor invariance yielded a good model fit for pretest – posttest and posttest – follow-up test (see Table S4 in the Online Supplement), indicating sound measurement properties for the scales.

**Online Supplement S4: Results of the Models Using an Alternative Model Specification (Grand-mean Centering Individual Student Level Predictors Instead of Group-mean Centering)**

**Program Effectiveness for Aggressive Behavior and Victimization**

In order to investigate program effectiveness, a multilevel analysis with change in aggressive behavior and victimization as dependent variables and *intervention* (0 = control group, 1 = intervention group) as a predictor on the class level (Model 1) were conducted. The results revealed statistically non-significant *intercepts* for the change in aggressive behavior (*b* = 0.031, *p* = .451) and in victimization (*b* = -0.021, *p* = .674), indicating that the control group’s aggressive behavior and victimization did not change between pre- and posttest. The predictor *intervention* was statistically significant for the change in victimization (*b* = -0.316, *p* < .001), but not statistically significant for the change in aggressive behavior (*b* = -0.037, *p* = .502). Hence, there was a decrease in victimization between pre- and posttest in the intervention group. These results indicate program effectiveness for victimization, but not for aggressive behavior.

As a next step, program effectiveness controlling for several covariates on the individual and class level assessed at pretest (Model 2, see Table S8) was re-estimated. More specifically, on the individual level, age, gender (0 = girls, 1 = boys), and aggressive behavior and victimization at pretest were included. All metric covariates were centered at the grand mean. On the class level, class climate and ethnic diversity were included as covariates, which were both centered at the grand mean. For the change in aggressive behavior, *aggressive behavior at pretest* (*b* = -0.401, *p* < .001), *victimization at pretest* (*b* = -0.081, *p* = .048) and *gender* (*b* = 0.145, *p* = .003) were statistically significant. This means that, controlling for all other covariates in the model, the higher one’s aggressive behavior and victimization at pretest, the stronger the decrease in aggressive behavior from pre- to posttest. In addition, boys experienced a stronger increase in aggressive behavior compared to girls. Regarding changes in victimization, the covariate *victimization at pretest* (*b* = -0.430, *p* < .001) was statistically significant, indicating that the higher one’s victimization at pretest, the stronger the decrease in victimization from pre- to posttest, controlling for all other covariates in the model. All other covariates were statistically non-significant.

The *intercept* for change in aggressive behavior (*b* = -0.063, *p* = .200) and change in victimization (*b* = -0.097, *p* = .096) were both statistically non-significant. The intervention effect was still present for victimization controlling for the covariates (predictor *intervention*, *b* = -0.241, *p* = .001), indicating a decrease in victimization in the intervention group between pre- and posttest. Regarding changes in aggressive behavior, *intervention* was still statistically non-significant (*b* = -0.008, *p* = .890), indicating no change in the intervention group between pre- and posttest. Overall, the results of the analyses controlling for covariates at the individual and class level demonstrated the effectiveness of the intervention regarding victimization, but not aggressive behavior.

**Program Sustainability for Aggressive Behavior and Victimization**

The results revealed statistically insignificant *intercepts* for the change in aggressive behavior (*b* = -0.030, *p* = .495) and the change in victimization (*b* = -0.084, *p* = .348), indicating no change in aggressive behavior or victimization in the control group (Model 1). However, *intervention* was statistically significant for the change in aggressive behavior (*b* = -0.260, *p* < .001), indicating reduced aggressive behavior after the posttest in the intervention group (sleeper effect). For changes in victimization, *intervention* was statistically non-significant (*b* = -0.108, *p* = .357), indicating a similar trend in both groups after the posttest. This means that program’s effects were sustained.

Furthermore, program sustainability was investigated while controlling for the same covariates at the individual and class level as for program effectiveness (Model 2, see Table S9). Regarding changes in aggressive behavior, *aggressive behavior at posttest* was statistically significant (*b* = -0.366, *p* < .001), indicating that the higher one’s aggressive behavior at posttest, the stronger the decrease in aggressive behavior from posttest to follow-up. Regarding changes in victimization, *aggressive behavior at posttest* (*b* = 0.184, *p* = .013) and *victimization at posttest* (*b* = -0.382, *p* < .001) were statistically significant: The higher one’s aggressive behavior at posttest, the stronger the increase in victimization, while the higher the victimization at posttest, the stronger the decrease in victimization from post-test to follow-up (controlling for all other covariates in the model). All other covariates were statistically non-significant. The *intercepts* for the change in aggressive behavior (*b* = 0.16, *p* = .734) and for the change in victimization (*b* = 0.014 *p* = .819) were statistically non-significant, thus revealing no changes in the control group after the posttest, controlling for covariates. Moreover, *intervention* was statistically significant for the change in aggressive behavior (*b* = -0.325, *p* < .001) and the change in victimization (*b* = -0.239, *p* = .015). These results reveal that there was a decrease in aggressive behavior and victimization in the intervention group after the posttest. In sum, the results demonstrate a sleeper effect of the intervention on aggressive behavior and sustainability of the intervention effect on victimization, while controlling for several covariates at the individual and class level.

**Class-level Moderators of Effectiveness**

In order to investigate class-level moderators of program effectiveness, we estimated a model including the interaction effects *intervention x class climate at pretest* and *intervention x ethnic diversity* on the class level (see Table S8, Model 3). For changes in aggressive behavior, *class climate* *at pretest* was statistically significant (*b* = -0.444, *p* = .012), indicating that the more positive the class climate in the control group, the stronger the decrease in aggressive behavior in the control group, controlling for all other covariates in the model. However, we did not find a statistically significant interaction effect, meaning that no moderation effect of class climate on effectiveness occurred. Regarding changes in victimization, *intervention* (*b* = -0.254, *p* < .001), *class climate at pretest* (*b* = -0.587, *p* = .001) and the interaction term *intervention x class climate* *at pretest* (*b* = -0.656, *p* = .002) were statistically significant. These findings reveal program effectiveness at average levels of class climate and ethnic diversity, and show that the more positive the class climate in the control group, the stronger the decrease in victimization in the control group, controlling for all other covariates in the model. However, the statistically significant interaction effect indicates that this positive effect of class climate on changes in victimization was not present in the intervention group; instead, a more positive class climate at pretest contributed to a smaller intervention effect. All other potential class-level moderators and interaction terms were statistically non-significant.

**Class-level Moderators of Sustainability**

In order to investigate class-level moderators of program sustainability, we estimated a model including the interaction effects *intervention x class climate* *at posttest* and *intervention x ethnic diversity* on the class level (see Table S9, Model 3). For changes in aggressive behavior, *intervention* (*b* = -0.272, *p* < .001), the interaction terms *intervention x class climate at posttest* (*b* = -0.296, *p* = .042) and *intervention* *x* *ethnic diversity* (*b* = -0.760, *p* = .023) were found to be statistically significant. These results indicated an intervention effect at average levels of class climate and ethnic diversity, and showed that the higher the positive class climate and ethnic diversity, the stronger the decrease in aggressive behavior in the intervention group (i.e., larger intervention effect). Regarding victimization, the main effects *class climate at posttest* (*b* = 0.290, *p* = .044) and *ethnic diversity* (*b* = 1.118, *p* = .017) as well as interaction effects *intervention x class climate* *at posttest* (*b* = -0.654, *p* = .001) and *intervention x ethnic diversity* (*b* = -1.966, *p* = .001) were statistically significant. These findings showed that (a) the higher the positive class climate and ethnic diversity, the stronger the increase in victimization in the control group, while (b) the higher the class climate and ethnic diversity, the stronger the decrease in victimization in the intervention group (i.e., larger intervention effect).

**Individual-level Moderators of Effectiveness**

We investigated individual-level moderators of program effectiveness in two steps.

First, we tested the variability of the slope parameters (i.e., random slopes) between classes of all covariates on the individual level using a series of deviance tests. The results revealed that for changes in aggressive behavior, there was a random slope effect for *aggressive behavior at pretest* ((1) = 6.258, *p* = .006), *victimization at pretest* ($\chi^{2}$(1) = 22.922, *p* < .001), and *gender* ($\chi^{2}$(1) = 3.172, *p* = .038). For changes in victimization, there was a random slope effect for *aggressive behavior at pretest* ($\chi^{2}$(1) = 8.278, *p* = .002), *victimization at pretest* ($\chi^{2}$(1) = 63.038, *p* < .001), and *gender* ($\chi^{2}$(1) = 3.056, *p* = .040).

Second, we predicted the variability between classes of the slope parameters for the covariates found significant in Step 1 using the predictor *intervention* (i.e., a cross-level interaction). The cross-level interaction *intervention x covariate* captures the moderating effect of an individual-level covariate on program effectiveness. For changes in aggressive behavior (see Table S8, Model 4), the cross-level interactions *intervention x* *aggressive behavior at pretest* (*b* = -0.539, *p* < .001) and *intervention* *x* *victimization at pretest* (*b* = 0.471, *p* < .001) were statistically significant, indicating that the higher one’s aggressive behavior at pretest, the stronger the decrease in aggressive behavior among students in the intervention group (i.e., larger intervention effect), and the higher the victimization at pretest, the smaller the decrease in aggressive behavior among students in the intervention group (i.e., smaller intervention effect), controlling for all other covariates. Regarding changes in victimization, the cross-level interaction *intervention x victimization at pretest* (*b* = -0.235, *p* < .001) was statistically significant, indicating that the higher the victimization at pretest, the stronger the decrease in victimization among students in the intervention group (i.e., larger intervention effect). All other cross-level interactions for changes in aggressive behavior and victimization were statistically non-significant (see Table S8, Model 4).

**Individual-level Moderators of Sustainability**

In order to investigate individual-level moderators of program sustainability, we first tested random effects of the slope parameters for all covariates on the individual level. Deviance tests indicated that for the change in aggressive behavior, there was a random slope effect for *aggressive behavior at posttest* ($\chi^{2}$(1) = 17.694, *p* < .001) and *gender* ($\chi^{2}$(1) = 3.646, *p* = .028). For the change in victimization, we obtained a random slope effect for *victimization at posttest* ($\chi^{2}$(1) = 2.762, *p* = .048).

In the second step, we predicted the variability of the slope parameters for the covariates found to be statistically significant using the predictor *intervention* (i.e., we estimated a cross-level interaction). For the change in aggressive behavior (see Table S9, Model 4), the cross-level interaction *intervention x* *aggressive behavior at posttest* (*b* = 0.339, *p* < .001) was statistically significant, indicating that the higher one’s aggressive behavior at posttest, the smaller the decrease in aggressive behavior in the intervention group (i.e., smaller intervention effect), controlling for all other covariates. Regarding changes in victimization, the cross-level interaction *intervention x victimization at posttest* (*b* = 0.320, *p* < .001) was statistically significant, controlling for all other covariates. This significant cross-level interaction indicated that the higher the victimization at posttest, the smaller the decrease in victimization in the intervention group (i.e., smaller intervention effect).
