# Supplementary material for: Understanding for Which Students and Classes a Socio-Ecological Aggression Prevention Program Works Best: Testing Individual Student and Class Level Moderators
Source: J Youth Adolesc. 2021 Dec 18;51(2):225–43. doi: 10.1007/s10964-021-01553-6 (PMC8828596; doi:10.1007/s10964-021-01553-6)
Supplement: Supplementary file 2 — Supplementary Information [file 10964_2021_1553_MOESM2_ESM.docx]

Table S1

*Patterns of Missing Data across Pre-, Post- and Follow-up test*

| Pretest | Posttest | Follow-up test | Missing Combined Sample *n* (%)  (N=2,078) | Missing Pre-Post Sample *n* (%)  (N=2,042) | Missing Post-Follow-Up Sample *n* (%)  (N=659) |
| --- | --- | --- | --- | --- | --- |
| 0 | 0 | 0 | no parental consent | | |
| 0 | 0 | 1 | 36 (1.7%) | - | 36 (5.5%) |
| 0 | 1 | 0 | 293 (14.1%) | 293 (14.3%) | 24 (3.6%) |
| 0 | 1 | 1 | 110 (5.3%) | 110 (5.4%) | 102 (15.5%) |
| 1 | 0 | 0 | 475 (22.9%) | 475 (23.3%) | 75 (11.4%) |
| 1 | 0 | 1 | 40 (1.9%) | 40 (2.0%) | 27 (4.1%) |
| 1 | 1 | 0 | 742 (35.7%) | 742 (36.3%) | 60 (9.1%) |
| 1 | 1 | 1 | 382 (18.4%) | 382 (18.7%) | 335 (50.8%) |

*Note*. 0 = wave nonresponse, 1 = wave response; *n* (%) Missing Combined Sample = frequency and percentage of missing data in the combined sample (N = 2,078); *n* (%) Missing Pre-Post Sample = frequency and percentage of missing data in the pre-post sample (N = 2,042); *n* (%) Missing Post-Follow-Up Sample = frequency and percentage of missing data in the post-follow up sample (N = 659).

Table S2

Missing Data Analysis: Differences Between Participants with Complete Data and Participants with Wave Nonresponse at Pretest

|  | Complete Data  (*n* = 1,127) | |  | Wave Nonresponse  (*n* = 512) | | *t* | *df* | *p* | Cohenꞌs *d* |
| --- | --- | --- | --- | --- | --- | --- | --- | --- | --- |
| Variable | *M* | *SD* |  | *M* | *SD* |  |  |  |  |
| Aggressive behavior | 0.41 | 0.47 |  | 0.48 | 0.53 | 2.671 | 880.76 | <.01 | 0.12 |
| Victimization | 0.59 | 0.65 |  | 0.70 | 0.76 | 2.803 | 859.36 | <.01 | 0.12 |

*Note*. Welch tests were used to investigate differences at pretest.

Table S3

Missing Data Analysis: Differences Between Participants with Complete Data and Participants with Wave Nonresponse at Posttest

|  | Complete Data  (*n* = 437) | |  | Wave Nonresponse  (*n* = 85) | | *t* | *df* | *p* | Cohenꞌs *d* |
| --- | --- | --- | --- | --- | --- | --- | --- | --- | --- |
| Variable | *M* | *SD* |  | *M* | *SD* |  |  |  |  |
| Aggressive behavior | 0.43 | 0.54 |  | 0.48 | 0.56 | 0.678 | 112.68 | .499 | 0.08 |
| Victimization | 0.51 | 0.62 |  | 0.49 | 0.71 | -0.212 | 109.83 | .832 | 0.03 |

*Note*. Welch tests were used to investigate differences at posttest.

Table S4

*Model Fit of the Measurement Models Assuming Strong Invariance*

|  | Pretest - Posttest | | | | |  | Posttest - Follow-up test | | | | |
| --- | --- | --- | --- | --- | --- | --- | --- | --- | --- | --- | --- |
|  | $\chi^{2}$ | *df* | *p* | CFI | RMSEA |  | $\chi^{2}$ | *df* | *p* | CFI | RMSEA |
| Aggressive behavior | 1060.78 | 641 | <.001 | .983 | .025 |  | 857.37 | 639 | <.001 | .968 | .032 |
| Victimization | 1158.53 | 641 | <.001 | .976 | .028 |  | 807.44 | 641 | <.001 | .977 | .028 |
| Class climate | 251.47 | 65 | <.001 | .969 | .053 |  | 171.59 | 65 | <.001 | .963 | .070 |

Table S5

*Bivariate Correlation Coefficients for Scales Assessing Aggressive Behavior and Victimization: Effectiveness Data (Pretest and Posttest)*

| Variable | 3. | 4. | 5. | 6. | 7. | 8. | 9. | 10. | 11. | 12. | 13. | 14. |
| --- | --- | --- | --- | --- | --- | --- | --- | --- | --- | --- | --- | --- |
| 1. Bullying Perpetration, Pretest |  |  |  |  |  |  |  |  |  |  |  |  |
| 2. Physical Aggression, Pretest | **.661** |  |  |  |  |  |  |  |  |  |  |  |
| 3. Relational Aggression, Pretest | **.653** | **.669** |  |  |  |  |  |  |  |  |  |  |
| 4. Bullying Perpetration, Posttest | **.310** | .300 | **.294** |  |  |  |  |  |  |  |  |  |
| 5. Physical Aggression, Posttest | **.259** | **.285** | **.234** | **.588** |  |  |  |  |  |  |  |  |
| 6. Relational Aggression, Posttest | **.223** | **.213** | **.228** | **.538** | **.768** |  |  |  |  |  |  |  |
| 7. Bullying Victimization, Pretest | **.522** | **.387** | **.436** | .167 | .117 | .083 |  |  |  |  |  |  |
| 8. Physical Victimization, Pretest | **.514** | **.415** | **.400** | .150 | .140 | .081 | **.661** |  |  |  |  |  |
| 9. Relational Victimization, Pretest | **.492** | **.434** | **.501** | .197 | .143 | .111 | **.694** | **.584** |  |  |  |  |
| 10. Bullying Victimization, Posttest | **.162** | .170 | **.192** | **.453** | **.330** | **.325** | **.356** | **.198** | **.311** |  |  |  |
| 11. Physical Victimization, Posttest | **.164** | **.190** | **.156** | **.403** | **.329** | **.304** | **.270** | **.217** | **.249** | **.653** |  |  |
| 12. Relational Victimization, Posttest | **.149** | .147 | **.163** | **.440** | **.278** | **.328** | .224 | .126 | .260 | **.671** | **.594** |  |

*Note*. *N* = 2042 from 105 classes.

Statistically significant correlation coefficients at α = .05 are boldface.

Table S6

*Bivariate Correlation Coefficients for Scales Assessing Aggressive Behavior and Victimization: Sustainability Data (Posttest and Follow-up Test)*

| Variable | 1. | 2. | 3. | 4. | 5. | 6. | 7. | 8. | 9. | 10. | 11. | 12. |
| --- | --- | --- | --- | --- | --- | --- | --- | --- | --- | --- | --- | --- |
| 1. Bullying Perpetration, Posttest |  |  |  |  |  |  |  |  |  |  |  |  |
| 2. Physical Aggression, Posttest | **.478** |  |  |  |  |  |  |  |  |  |  |  |
| 3. Relational Aggression, Posttest | **.397** | **.732** |  |  |  |  |  |  |  |  |  |  |
| 4. Bullying Perpetration, Follow-up test | **.427** | **.304** | **.250** |  |  |  |  |  |  |  |  |  |
| 5 Physical Aggression, Follow-up test | **.335** | **.303** | **.289** | **.416** |  |  |  |  |  |  |  |  |
| 6. Relational Aggression, Follow-up test | **.275** | **.208** | **.284** | **.353** | **.630** |  |  |  |  |  |  |  |
| 7. Bullying Victimization, Posttest | **.351** | **.208** | **.183** | **.246** | .120 | .093 |  |  |  |  |  |  |
| 8. Physical Victimization, Posttest | **.320** | **.235** | **.191** | **.190** | **.184** | **.141** | **.638** |  |  |  |  |  |
| 9. Relational Victimization, Posttest | **.365** | **.176** | **.217** | **.234** | .093 | .140 | **.646** | **.572** |  |  |  |  |
| 10. Bullying Victimization, Follow-up test | **.311** | **.173** | **.152** | **.345** | **.203** | **.199** | **.492** | **.374** | **.332** |  |  |  |
| 11. Physical Victimization, Follow-up test | **.247** | **.168** | **.140** | **.299** | **.308** | **.280** | **.332** | **.399** | **.229** | **.528** |  |  |
| 12. Relational Victimization, Follow-up test | **.308** | **.156** | **.180** | **.283** | **.269** | **.334** | **.341** | **.334** | **.326** | **.616** | **.643** |  |

*Note*. *N* = 259 from 35 classes; triangle.
Statistically significant correlation coefficients at α = .05 are boldface.

Table S7

*Null Model: Variance Components, Intraclass Correlations and Model Summary*

|  | Posttest - Pretest | | |  | Follow-up test - Posttest | | |
| --- | --- | --- | --- | --- | --- | --- | --- |
|  | ΔAggressive Behavior |  | ΔVictimization |  | ΔAggressive Behavior |  | ΔVictimization |
| Variance components  Level 2 – Class  Level 1 – Individual | 0.020  0.898 |  | 0.056  1.113 |  | 0.024  0.285 |  | 0.053  0.736 |
| Intraclass correlation  95% CI | 0.021  [0.000, 0.045] |  | 0.048  [0.022, 0.075] |  | 0.078  [0.016, 0.141] |  | 0.067  [0.000, 0.129] |
| Model summary  Deviance  AIC | 10053.77  10069.77 | | |  | 2519.854  2535.853 | | |

Table S8

*Alternative Model Specifications: Multilevel Modeling Results: Effectiveness (Posttest – Pretest) for Aggressive Behavior and Victimization Using Grand-Mean Centering for Individual Student Level Predictors*

|  | Posttest - Pretest | | | | | | | | | | | | | | | | | |
| --- | --- | --- | --- | --- | --- | --- | --- | --- | --- | --- | --- | --- | --- | --- | --- | --- | --- | --- |
|  | Model 2 | | | | | |  | Model 3 | | | | |  | Model 4 | | | | |
|  | ΔAggressive  behavior | |  | | ΔVictimization | |  | ΔAggressive behavior | |  | ΔVictimization | |  | ΔAggressive behavior | |  | ΔVictimization | |
| Coefficient | *b* | SE |  | | *b* | SE |  | *b* | SE |  | *b* | SE |  | *b* | SE |  | *b* | SE |
| Level 1 – Individual  Intercept  Aggressive behavior at pretest  Victimization at pretest  Age at pretest  Gender (0 = female, 1 = male) | -0.063  **-0.401**  **-0.081**  0.006  **0.145** | 0.049  0.051  0.041  0.027  0.048 |  | -0.097  -0.088  **-0.430**  -0.022  0.049 | | 0.012  0.049  0.046  0.031  0.053 |  | -0.060  **-0.402**  -0.079  0.006  **0.147** | 0.049  0.051  0.041  0.027  0.048 |  | -0.078  -0.087  **-0.427**  -0.021  0.051 | 0.060  0.049  0.046  0.031  0.053 |  | -0.056  0.008  **-0.455**  -0.002  0.086 | 0.052  0.092  0.081  0.026  0.079 |  | -0.069  -0.081  **-0.243**  -0.017  0.056 | 0.070  0.110  0.100  0.030  0.104 |
| Level 2 – Class  Intervention (0 = control, 1 = intervention)  Class climate at pretest  Ethnic diversity  Intervention x class climate at pretest  Intervention x ethnic diversity  Intervention x aggressive behavior at pretest  Intervention x victimization at pretest  Intervention x gender | -0.008  -0.163  -0.017 | 0.060  0.100  0.125 |  | **-0.241**  -0.115  -0.171 | | 0.071  0.097  0.139 |  | -0.011  **-0.444**  0.461  0.365  -0.582 | 0.058  0.177  0.299  0.212  0.330 |  | **-0.254**  **-0.587**  0.287  **0.656**  -0.500 | 0.070  0.178  0.317  0.214  0.349 |  | -0.028  **-0.405**  0.291  0.298  -0.396  **-0.539**  **0.471**  0.078 | 0.068  0.153  0.274  0.197  0.310  0.105  0.091  0.095 |  | **-0.255**  **-0.463**  0.337  **0.443**  **-0.555**  -0.028  **-0.235**  -0.012 | 0.086  0.175  0.307  0.218  0.340  0.121  0.111  0.118 |
| Variance components  Level 1 – individual  Level 2 – class  Slope aggressive behavior at pretest  Slope victimization at pretest  Slope gender | 0.691  0.031 |  |  | | 0.823  0.036 |  |  | 0.691  0.027 |  |  | 0.823  0.027 |  |  | 0.660  0.025  0.007  0.000  0.001 |  |  | 0.785  0.026  0.003  0.008  0.012 |  |
| Model summary  Deviance  AIC | 9139.652  91.83.653 | | | | | |  | 9124.070  9176.071 | | | | |  | 8846.014  8922.013 | | | | |

*Note*. Unstandardized coefficients.

Gender is coded as 0 = females and 1 = males; Intervention is coded as 0 = control group and 1 = intervention group.

Statistically significant coefficients at α = .05 are shown in boldface.

Table S9

*Alternative Model Specifications: Multilevel Modeling Results: Sustainability (Follow-up test - Posttest) for Aggressive Behavior and Victimization Using Grand-Mean Centering for Individual Student Level Predictors*

|  | Follow-up test - Posttest | | | | | | | | | | | | | | | | |
| --- | --- | --- | --- | --- | --- | --- | --- | --- | --- | --- | --- | --- | --- | --- | --- | --- | --- |
|  | Model 2 | | | | |  | Model 3 | | | | |  | Model 4 | | | | |
|  | ΔAggressive behavior | |  | ΔVictimization | |  | ΔAggressive behavior | |  | ΔVictimization | |  | ΔAggressive behavior | |  | ΔVictimization | |
| Coefficient | *b* | SE |  | *b* | SE |  | *b* | SE |  | *b* | SE |  | *b* | SE |  | *b* | SE |
| Level 1 – Individual  Intercept  Aggressive behavior at posttest  Victimization at posttest  Age at posttest  Gender (0 = girls, 1 = boys) | 0.016  **-0.366**  0.052  0.017  -0.028 | 0.048  0.060  0.028  0.027  0.043 |  | 0.014  **0.184**  **-0.382**  -0.015  -0.058 | 0.063  0.074  0.042  0.039  0.062 |  | 0.000  **-0.373**  **0.055**  0.024  -0.029 | 0.059  0.060  0.028  0.027  0.046 |  | -0.035  **0.169**  **-0.374**  -0.002  -0.061 | 0.061  0.072  0.041  0.039  0.067 |  | 0.027  **-0.500**  0.039  0.005  -0.020 | 0.067  0.055  0.024  0.028  0.036 |  | -0.031  0.128  **-0.491**  -0.020  -0.059 | 0.054  0.070  0.036  0.041  0.067 |
| Level 2 – Class  Intervention (0 = control, 1 = intervention)  Class climate at posttest  Ethnic diversity  Intervention x class climate at posttest  Intervention x ethnic diversity  Intervention x aggressive behavior at posttest  Intervention x victimization at posttest  Intervention x gender | **-0.325**  0.116  -0.106 | 0.077  0.095  0.165 |  | **-0.239**  0.224  -0.078 | 0.098  0.154  0.348 |  | **-0.272**  0.157  0.359  **-0.296**  -**0.760** | 0.071  0.126  0.277  0.145  0.334 |  | -0.106  **0.290**  **1.118**  **-0.654**  **-1.966** | 0.088  0.145  0.467  0.206  0.570 |  | **-0.283**  0.232  0.105  **-0.316**  -0.509  **0.339**  -0.054 | 0.075  0.126  0.318  0.155  0.353  0.062  0.065 |  | -0.112  0.224  0.896  **-0.515**  **-1.613**  **0.320** | 0.087  0.125  0.418  0.203  0.514  0.056 |
| Variance components  Level 1 – individual  Level 2 – class  Slope aggressive behavior at posttest  Slope victimization at posttest  Slope gender | 0.224  0.003 |  |  | 0.605  0.015 |  |  | 0.222  0.001 |  |  | 0.802  0.061 |  |  | 0.194  0.000  0.004  0.009 |  |  | 0.563  0.001  0.000 |  |
| Model summary  Deviance  AIC | 2044.830  2088.730 | | | | |  | 2026.274  2078.275 | | | | |  | 1933.130  1997.130 | | | | |

*Note*. Unstandardized coefficients.

Gender is coded as 0 = females and 1 = males; Intervention is coded as 0 = control group and 1 = intervention group.

Statistically significant coefficients at α = .05 are shown in boldface.
